# Azathioprine-Associated Acute Pancreatitis Followed by a Probable Autoimmune Hepatitis Flare After Thiopurine Withdrawal: A Case Report

**DOI:** 10.7759/cureus.102162

**Published:** 2026-01-23

**Authors:** Emmanuel Marfo, Vicki Oladoyin, Julia Liu

**Affiliations:** 1 Internal Medicine, Morehouse School of Medicine, Atlanta, USA; 2 Internal Medicine, Grady Memorial Hospital, Atlanta, USA; 3 Gastroenterology, Morehouse School of Medicine, Atlanta, USA; 4 Gastroenterology, Grady Memorial Hospital, Atlanta, USA

**Keywords:** autoimmune hepatitis flare, azathioprine-induced pancreatitis, drug-induced acute pancreatitis, immunosuppression discontinuation, thiopurine withdrawal

## Abstract

Azathioprine is commonly used in the management of inflammatory bowel disease and autoimmune hepatitis (AIH). Acute pancreatitis is a recognized idiosyncratic adverse effect that necessitates drug discontinuation. In patients with immune-mediated liver disease, abrupt withdrawal of immunosuppression may predispose to disease flares. We present a case of probable azathioprine-associated acute pancreatitis, followed by biochemical hepatitis consistent with a probable AIH flare after thiopurine cessation. This case highlights the clinical challenge of balancing adverse drug reactions with maintenance of disease control in patients requiring chronic immunosuppression.

## Introduction

Thiopurines, including azathioprine and 6-mercaptopurine, remain cornerstone immunomodulators in inflammatory bowel disease (IBD) and are guideline-supported first-line agents for autoimmune hepatitis (AIH), typically administered in combination with corticosteroids [1,2]. The American Association for the Study of Liver Diseases recommends prednisone 20-60 mg daily combined with azathioprine 50-150 mg daily for induction therapy in AIH, with subsequent dose reduction to maintain biochemical remission [1]. Similarly, in IBD, the American College of Gastroenterology recommends azathioprine at doses of 1.5-2.5 mg/kg/day and 6-mercaptopurine at doses of 0.75-1.5 mg/kg/day as steroid-sparing maintenance agents, though their role has evolved with the availability of biologic therapies [3,4].

While many adverse effects of azathioprine are dose-dependent, including myelosuppression, hepatotoxicity, and vascular endothelial lesions related to thiopurine metabolite accumulation, acute pancreatitis represents an idiosyncratic, dose-independent reaction [5-7]. The incidence of azathioprine-induced pancreatitis (AIP) in IBD populations ranges from 3% to 7.5%, with the highest rates reported in Crohn's disease [8-11]. Large-scale pharmacovigilance data demonstrate a particularly strong association between thiopurines and acute pancreatitis when used for Crohn's disease (reporting odds ratio 34.61) compared to ulcerative colitis (reporting odds ratio 8.94) [10].

AIP typically occurs early after drug initiation, with a median onset of four weeks and 91% of cases developing within the first three months of therapy [8,9,12]. The clinical course is characteristically mild, with all patients in multiple large series experiencing complete resolution upon medication cessation and no reported complications [8,9]. Genetic susceptibility plays a significant role, as the HLA-DQA102:01-HLA-DRB107:01 haplotype (rs2647087) confers substantially elevated risk, with heterozygous carriers having a 9% risk and homozygous variant carriers a 14-17% risk of developing AIP [2,5,12].

However, discontinuation of thiopurines in patients with immune-mediated diseases may precipitate disease flares if alternative immunosuppression is not promptly instituted. In IBD patients who discontinue azathioprine while in clinical remission, relapse rates reach 23% in Crohn's disease and 12% in ulcerative colitis at 12 months, increasing to 39% and 26% respectively at 24 months [7]. A recent meta-analysis of 3,057 patients demonstrated a pooled relapse rate of 32.5% following azathioprine withdrawal, with ulcerative colitis patients experiencing higher relapse rates (41.3%) than Crohn's disease patients (24.7%) [8]. Similarly, in AIH, withdrawal of azathioprine after achieving remission carries substantial risk of disease reactivation, necessitating careful consideration of alternative immunosuppressive strategies [1,2].

This clinical scenario presents a significant management dilemma: balancing the imperative to discontinue a medication causing an idiosyncratic adverse reaction against the risk of disease flare in conditions requiring sustained immunosuppression. We report a case illustrating this therapeutic challenge and review evidence-based approaches to managing AIP in patients with immune-mediated diseases, which was clinically presumed due to her presentation.

## Case presentation

A 44-year-old woman with a history of IBD managed with guselkumab and a prior clinical diagnosis of AIH based on elevated smooth muscle autoimmune titers (>1:640) and prior biochemical response to immunosuppression presented with several days of epigastric pain radiating to the back, associated with nausea and vomiting. She had recently been treated with azathioprine 100 mg daily, which she started approximately three months prior to her admission. See Table 1 for her initial presenting labs.

**Table 1:** Initial Presentation of Acute Pancreatitis ULN: upper limit of normal

Laboratory Test	Value	Reference Range	Interpretation
Lipase	999 U/L	60 U/L	Elevated (>16 × ULN)
Triglycerides	68 mg/dL	149 mg/dL	Normal
Calcium	8.4 mg/mL	10.3 mg/dL	Normal

Cross-sectional imaging of the abdomen and pelvis showed no acute pancreatic or biliary abnormalities. The patient met two of three diagnostic criteria for acute pancreatitis according to the revised Atlanta classification: characteristic abdominal pain and serum lipase greater than three times the upper limit of normal [1]. Given the temporal association with azathioprine use (median onset four weeks, with 91% of cases occurring within the first three months) and absence of an alternative clear etiology, with the lack of alcohol use, lack of biliary obstructions on imaging, normal calcium levels, and normal triglycerides, azathioprine-associated acute pancreatitis was considered the most likely diagnosis, and the medication was discontinued. Due to her imaging being normal, her pancreatitis is classified as mild pancreatitis. Her symptoms improved with supportive care, and she was discharged.

Readmission - suspected AIH flare

The patient was readmitted with abdominal pain, jaundice, and overall malaise 19 days after being discharged. The patient's significant labs can be seen in Table 2.

**Table 2:** Key Laboratory Results ULN: upper limit of normal; AST: aspartate aminotransferase; ALT: alanine aminotransferase

Laboratory Test	Value	Reference Range	Interpretation	Baseline
Total Bilirubin	4.6 mg/dL	0.1-1.2 mg/dL	Markedly elevated	1.2 mg/dL
AST	672 U/L	10-40 U/L	Markedly elevated (>16 × ULN)	19 U/L
ALT	476 U/L	7-56 U/L	Markedly elevated (>8 × ULN)	20 U/L
White Blood Cell Count	6.1 x 10^9^/L	4.5-11.0 × 10^9^/L	Normal	4.28 × 10^9^/L
Hemoglobin	10.0 g/dL	12.0-16.0 g/dL (female)	Low	11.6

An infectious evaluation, including stool studies, blood cultures, respiratory cultures, viral hepatitis labs (A, B, C, and E), and CT chest and abdominal imaging, did not identify an acute infectious process.

Given her history of autoimmune liver disease and recent cessation of immunosuppressive therapy, the presentation was most consistent with a probable AIH flare. At the time of presentation, the patient did not consume any alcohol and had not started any new medications since her last admission. She had an International Autoimmune Hepatitis Group (IAIHG) score of 8, an elevated IgG of 2006 mg/dL, and a history of a negative L‑K microsomal antibody test. Although a liver biopsy was not performed, prior serologic results - autoantibody titers exceeding 1:640 - and a prompt response to corticosteroids supported an immune‑mediated process. She was started on prednisone 40 mg daily, which led to improvement in transaminase levels during hospitalization (Table 3). She was discharged on a steroid taper with close gastroenterology follow‑up and plans to transition to an alternative long‑term immunosuppressive regimen.

**Table 3:** Relevant Laboratory Results at Discharge ULN: upper limit of normal; AST: aspartate aminotransferase; ALT: alanine aminotransferase

Laboratory Test	Value	Reference Range	Interpretation
Total Bilirubin	4.6 mg/dL	0.1-1.2 mg/dL	Markedly elevated
AST	277 U/L	10-40 U/L	Markedly elevated (>16 × ULN)
ALT	317 U/L	7-56 U/L	Markedly elevated (>8 × ULN)

## Discussion

AIP is a well-described but unpredictable adverse drug reaction. Unlike other thiopurine toxicities, it is not dose-dependent and does not correlate with thiopurine methyltransferase (TPMT) or NUDT15 variants, which primarily predict myelosuppression risk [2,9,11,12]. While TPMT and NUDT15 genetic variants are strongly associated with bone marrow toxicity through altered thiopurine metabolism, AIP appears to be genetically influenced by the HLA-DQA102:01-HLA-DRB107:01 haplotype (rs2647087), with heterozygous carriers having a 9% risk and homozygous variant carriers a 14-17% risk [2,5,12]. This patient was specifically tested for the TMPT and NUDT15 variations, but was not tested for HLA variants.

The precise mechanism of AIP remains incompletely understood, though both immune-mediated and direct toxic pathways are suspected [2,10,12]. Recent mechanistic studies have identified that azathioprine impairs pancreatic ductal bicarbonate and chloride secretion through inhibition of RAC1 activity, leading to diminished CFTR expression at the apical plasma membrane [12]. Additionally, genome-wide association studies have identified novel genetic associations with RAB19 (rs2948386) and predicted pancreatic expression of SERPINB9P1, suggesting multiple pathophysiologic mechanisms may contribute to this idiosyncratic reaction [10].

The diagnosis of drug-induced pancreatitis is one of exclusion, requiring fulfillment of standard acute pancreatitis criteria (typically two of three: characteristic abdominal pain, serum lipase or amylase at least three times the upper limit of normal, and characteristic imaging findings) while systematically excluding other etiologies [13,14]. However, in clinical practice, complete exclusion of all alternative etiologies may not always be feasible, and the evidence base for drug-induced pancreatitis associations is often tenuous, with incomplete exclusion of non-drug causes reported in most case series [13]. In this case, the temporal relationship between azathioprine exposure and symptom onset, combined with clinical improvement following drug discontinuation, supported a probable diagnosis.

A key teaching point from this case is the substantial risk of disease flare following abrupt withdrawal of immunosuppression, particularly in patients with autoimmune conditions such as AIH. Relapse occurs in 50-87% of adults with AIH after drug withdrawal, even in patients who have achieved biochemical normality for at least two years during treatment [1,2]. The majority of relapses (50%) occur within the first three months after drug withdrawal, with 90% occurring within 28 months [1]. Although the patient's AIH diagnosis was not biopsy-confirmed, her prior serologic findings and steroid responsiveness supported an immune-mediated process. The biochemical flare shortly after azathioprine discontinuation and subsequent improvement with corticosteroids further supported this interpretation.

This case underscores the importance of proactive immunosuppressive planning when thiopurines must be discontinued. Early initiation of bridging therapy with corticosteroids is essential to prevent disease flares [1]. For long-term management, steroid-sparing alternatives should be considered, including mycophenolate mofetil (MMF) or calcineurin inhibitors (cyclosporine, tacrolimus) [1,2,15,16]. Recent evidence suggests MMF combined with prednisolone may achieve higher rates of biochemical remission (56.4% vs. 29.0%) with superior tolerability compared to azathioprine in treatment-naive AIH patients [15]. For patients intolerant to azathioprine, MMF demonstrates an 82% response rate as second-line therapy [1]. Calcineurin inhibitors represent another alternative, though their use is limited by potential toxicities including hypertension, renal insufficiency, and increased infection risk [1,2]. Thus, choosing which immunosuppressive agent is something that should be discussed with the patient and is an individualized choice depending on their other health co-morbidities.

Multidisciplinary collaboration between gastroenterology and hepatology is critical for patients with overlapping immune‑mediated diseases. Such coordinated care enables clinicians to optimize immunosuppressive regimens and reduce the risk of disease flares during transitions between therapies. Understanding these risks is essential, as poorly managed medication changes can lead to significant clinical setbacks. By anticipating potential adverse effects, clinicians can develop individualized treatment plans that minimize withdrawal‑related complications and support more stable long‑term disease control. A potential plan could be to taper the patient with glucocorticoid steroids until their outpatient gastroenterology appointment.

## Conclusions

Azathioprine‑associated acute pancreatitis necessitates immediate discontinuation of the drug; however, doing so may place patients with autoimmune liver disease at heightened risk for disease flare if alternative immunosuppression is not promptly initiated. Clinicians should anticipate this vulnerability and employ early bridging therapy to preserve disease stability during the transition. This case underscores a broader, often underrecognized management challenge in patients with concurrent immune‑mediated conditions and highlights the importance of anticipatory strategies as a generalizable principle in complex immunosuppressive care.
